# Nurses' Experiences of Communicating With Families in Municipal Home Health Care

**DOI:** 10.1002/nop2.70117

**Published:** 2024-12-06

**Authors:** Elisabeth Bruce, Susanna Pusa, Karin Sundin

**Affiliations:** ^1^ Department of Nursing Umeå University Örnsköldsvik Sweden

**Keywords:** family, family nurse practitioners, health communication, home health nursing, nursing care, qualitative research

## Abstract

**Aim:**

To illuminate nurses' experiences of communicating with families in home health care.

**Design:**

A qualitative inductive approach.

**Background:**

An increasing number of ageing and sick people are being granted home health care. Nurses' duties involve caring for both patients and their families, which includes the important task of meeting and talking with them.

**Methods:**

Fourteen registered nurses working in home health care in Sweden participated in individual narrative semistructured interviews. The interviews were analysed with content analysis. To make sure all components of the study were clear, the Standards for Reporting Qualitative Research checklist was used as a guide.

**Result:**

Communication with families was viewed as a crucial action for building rapport to facilitate the involvement and support of families in difficult situations. The nurses highlighted the struggles they sometimes faced in communication when they found it difficult to reach the family. When the communication was supportive for the family, the nurses felt that they were empowering the family members to empower themselves.

**Conclusion:**

This study highlights the vital role of communication in home health care nursing. Effective communication fosters trust and enables nurses to meet family needs.

**Implications for the Profession:**

Effective communication between nurses and families in home health care is crucial from a health care perspective. Building reciprocal relationships fosters trust, enabling nurses to efficiently identify and address family needs and enhancing the quality of care.

**Patient or Public Contribution:**

No patient or public contribution.

## 1 Background

Worldwide, the number of persons aged over 60 years is increasing (World Health Organisation 2022). The demand for home health care (HHC) is also increasing, as older people with declining health continue to live in their own homes (National Board of Health and Welfare 2020). HHC is defined as medical procedures, physical therapy and nursing care given in ordinary homes, and is an option for those who require long‐term health care (National Board of Health and Welfare 2017). The composition of HHC teams can vary, and a scoping review has shown that there are unclear or absent definitions of the professions and roles included in these teams. However, the presence of nurses in HHC teams is crucial in providing care for patients at home, since nurses serve as the link between professionals, patients and families (Larsson et al. 2022).

When a person is granted HHC, their family, if they have one, is normally incorporated into their care in one way or another. Those family members who take on a greater role in caring for sick relatives at home are known as family caregivers (Schulz and Eden 2016). Caregiver burden has been reported as common among family members (Riffin et al. 2019). Family members not only contend with the distress and uncertainty associated with their loved one's illness, but also shoulder the responsibility for providing social support, coordination of activities and practical management (Howard et al. 2023). The pressures that family caregivers frequently place on themselves, along with perceived demands from patients, nurses and society, may lead to feelings of inadequacy (Brobäck and Berterö 2003; Eriksson and Svedlund 2006) and of ‘struggling alone’ (Howard et al. 2023). Family caregivers often want to care for patients at home, but may not always feel that they can handle this. Although they may feel guilty if they require a vacation from their caring duties, it is crucial that they take time for themselves to deal with daily life (Brobäck and Berterö 2003). According to Brobäck and Berterö (2003) and Lethin et al. (2016), family caregivers may believe that no one is concerned about how they are feeling. They may lack the time or energy for socialising, which results in their feeling socially alienated and losing interest in social activities (Eriksson and Svedlund 2006).

Given that families are those who know the patients best and are closest to the person in need of care, nurses often show positive attitudes regarding the importance of family involvement in nursing care, including the perceived appropriateness of involving the family in the care process (Cranley et al. 2022; Hagedoorn et al. 2020; Østergaard et al. 2020; Shamali et al. 2023). A reciprocal relationship between nurses and families could be crucial for reducing suffering and fostering health. The relevance of transition from patient‐centred care, where the nurse is perceived as the sole specialist, to family‐centred care, where all parties in the whole family are equally involved, has been discussed by Shajani and Snell (2022). The circumstances for good care are established by the nurse's effective communication with the patient and their family members; this includes conversations which allow verbal exchange of information or the sharing of ideas, emotions and experiences between two or more people (Kourkouta and Papathanasiou 2014). Well‐functioning communication between health care providers, patients and family members has been highlighted as a crucial facilitator for fostering relationships (Gregory et al. 2017).

Studies on communication with families are often related to specific interventions (e.g., Eltaybani et al. 2022) and/or implementation of specific conversation methods (Carlsson 2014; Pusa, Isaksson, and Sundin 2021), or focus on specific areas and disease states, such as palliative care in the home (e.g., Becqué et al. 2019), stroke (Wallengren et al. 2008) or dementia (Lethin et al. 2016). There is a lack of studies addressing nurses' experiences of conversations in general with families in HHC. Exploring this area is crucial because nurses and specialist nurses in HHC encounter a diverse range of patients and families. Understanding how nurses experience their interactions with families in HHC is essential to identify key aspects that can enhance communication practices. This understanding is vital for advancing HHC practices with families, ensuring that care is both effective and empathetic.

## 2 The Study

### 2.1 Aim

To illuminate nurses' experiences of communicating with families in HHC.

## 3 Methods

### 3.1 Design

This descriptive qualitative interview study used an inductive approach to illuminate nurses' experiences. The data were gathered using semistructured interviews for their flexibility in exploring participants' experiences and covering key topics (cf. Polit and Beck 2022) and analysed with qualitative content analysis to gain insight into the nurses' experiences of communicating with the families in HHC (Graneheim and Lundman 2004; Lindgren, Lundman, and Graneheim 2020). The Standards for Reporting Qualitative Research checklist (Appendix 1; O'Brien et al. 2014) served as the foundation for the study.

### 3.2 Study Setting and Sampling

The study setting was HHC in a municipality in northern Sweden. The municipal population has a slightly higher median age than the national average, contains both urban and rural areas and includes culturally diverse families. Before the study began, the municipality's director of HHC was contacted to request permission to conduct participant recruitment to the study at their unit, and to ask for assistance in the identification of nurses who met the inclusion criteria. As this was a nonprobability sampling process, all eligible nurses who met these criteria were asked if they would like to participate in the study. The nurses were also informed verbally and in writing of the purpose of the research, the implications of participation, the fact that participation was entirely voluntary and the right to withdraw from the study at any time and without explanation. All participants provided their written consent.

### 3.3 Inclusion Criteria

The inclusion criteria for this study were being a registered nurse or specialist nurse working during the daytime in HHC, and having a minimum of 2 years' experience as a nurse. This is because the nurses who work during the day had experience with meeting families regularly. A total of 15 potential participants were identified based on the inclusion criteria, but one refrained from participating in the interview process due to work‐related reasons.

### 3.4 Data Collection

Sociodemographic questions covering aspects such as age, gender, work experience and education level were asked prior to the interview. Data were gathered through narrative semistructured face‐to‐face individual interviews conducted by a single researcher (SP), who is a registered nurse with HHC experience. All participants chose to be interviewed at the university campus rather than at their places of employment. Each interview was audio recorded, lasted 18–40 min and was later transcribed verbatim. Although some interviews were brief, they provided rich data on the nurses' experiences. An interview guide comprising open‐ended questions was used. Two main questions were asked: ‘How do you feel about communicating with families in HHC?’ and ‘How would you like to communicate with families?’. During the interviews, probing questions such as ‘Can you tell me more?’ and ‘Can you explain in more detail?’ as well as ‘When?’, ‘Where?’ and ‘How?’ were used to elicit an even more thorough and comprehensive account.

### 3.5 Data Analysis

To interpret the data and gain knowledge about nurses' experiences of communicating with families while providing HHC, a qualitative content analysis was conducted (cf. Graneheim and Lundman 2004; Lindgren, Lundman, and Graneheim 2020). All authors (EB, SP and KS) actively participated in the analysis process. The transcribed interviews were read several times both separately and together to get a general sense of the text. The analytical process then started with step‐by‐step abstracting and interpreting of the text through two phases: decontextualisation (extraction of meaning units, condensing, coding) and recontextualisation (sorting codes into subcategories and categories). To logically follow the analysis from decontextualisation to recontextualisation, the analysis was carried out using an Excel spreadsheet with one column for each of the five steps in the analytical process: meaning units, condensations, codes, subcategories and categories.

The first phase of the analysis—decontextualisation—began with step 1, which involved segmenting the text into meaning units comprising paragraphs and/or words in the text that had the same content and responded to the aim of the study. Step 2 was concerned with eliminating extraneous words; the meaning units were condensed into shorter versions that still retained the original content. The condensed meaning units were then given codes in the third step, which meant marking each of them with a title that summarised its content. The recontextualisation phase began with step 4, grouping the codes by their shared characteristics and similarities. The grouped codes were then abstracted, interpreted into subcategories based on their content and given names that reflected their overall meaning. In step 5, the same process was used to create categories; here, the subcategories with similar content were grouped together, and by abstracting and interpreting their content, the categories were formulated and named to illustrate the overall content of the grouped subcategories. The analytical process is illustrated in an example in Table 1.

**TABLE 1:** Example of the analysis process.

Decontextualisation			Recontextualsation	
Meaning unit	Condensation	Code	Subcategory	Category
The most important thing is to build trust so that they genuinely want to and feel able to talk … because that is truly the most essential thing	Most important thing is to build trust so that they want to talk. That is absolutely essential	Creating trust	Building rapport	Becoming familiar with the family

### 3.6 Ethical Considerations

Our study was conducted in accordance with the Declaration of Helsinki (World Medical Association 2023). Before the interviews began, participants were required to provide both written and verbal consent. They were also informed that if they felt uncomfortable due to their participation, they could contact their workplace's Occupational Health Service. The decoded interview materials and the code list were stored separately, in a fireproof cabinet at the university. Confidentiality in the study was taken into consideration. It was also kept in mind that the participants might find their participation emotionally challenging when they narrated experiences that could make them perceive personal flaws in their professional role. The ethical approval of the study was obtained in 2014 from the Ethical Review Board (No 2014‐235‐31Ö), and data were collected at the end of 2018.

### 3.7 Rigour and Reflexivity

Trustworthiness (Graneheim and Lundman 2004; Lindgren, Lundman, and Graneheim 2020) was considered throughout the whole process of conducting the study. Credibility was enhanced by the fact that the study population comprised registered nurses who were actively working in HHC, by the use of interviews to collect the data and by the choice of qualitative content analysis as analytical method, which contributed to answering the purpose. Participation of all authors in the analysis and discussion of the most probable interpretation improved the confirmability, and the use of semistructured interviews conducted by just one person over a brief period improved the dependability.

The research team consists of three members. There was some slight variation in the researchers' preunderstanding of the context in which the studied phenomenon of communication took place. All authors are female registered nurses. The last author (KS) is an experienced researcher with an in‐depth understanding of the family‐centred approach to, among other things, HHC. The second author (SP) has both clinical and research experience of family‐centred communication in HHC. The first author (EB) has a research focus and a deeper understanding of family‐centred care within paediatric nursing. All authors sought to mitigate the influence of their preunderstanding during the interviews and data analysis. On the other hand, the prior knowledge of the researchers adds to the theoretical depth and breadth of the analysed data. Regarding transferability, detailed descriptions of the participants and the study's results enable an understanding of when the study findings can be applied to contexts beyond the present one. We believe that transferability of the results is possible in other contexts when nurses interact with families during health care meetings.

## 4 Findings

### 4.1 Characteristics of Participants

There was a total of 14 participants: thirteen women and one man. They ranged in age from 27 to 59, with an average age of 43 years. The majority had worked as a nurse for 13 years or longer (*n* = 9), while two nurses had worked for 8–12 years and three for 2–7 years. Regarding education, seven were registered nurses with specialist degrees in primary health care nursing, two were registered nurses with other specialist education in nursing care and five were registered nurses without a specialist degree in nursing.

## 5 Results

Analysis of the interviews resulted in three categories and seven subcategories (Table 2).

**TABLE 2:** Categories and subcategories.

Categories	Subcategories
Becoming familiar with the family	Building rapport
	Exploring the family's communication needs
Struggling to reach the family	Lack of understanding hinders effective communication
	Challenges in engaging the whole family
Empowering the family to empower themselves	Acknowledging the value of the family
	Conciliating strengths

### 5.1 Becoming Familiar With the Family

This category summarises the nurses' experiences of building rapport through family–nurse interactions and assessing the family's communication needs by exploring their preferences.

#### 5.1.1 Building Rapport

Building rapport encompassed communication and interpersonal relationships between nurse and family as well as within the family. The nurses believed that having positive and honest communication with the families strengthened their relationship. Engaging in conversations with families could benefit all parties involved—the families, the patients and the nurses. This practice not only strengthened the connections between the nurses and the family, but also fostered deeper bonds among family members. It went beyond mere communication, weaving a tapestry of rapport building and support. The nurses felt that their responsibility extended to cultivating an environment conducive to natural and relaxed communication, allowing families to feel acknowledged.

There should be an environment where family members feel that we are there for them and that they can bring up … both big and small matters with me. (Nurse 26)

They emphasised that having a delicate touch was crucial, as well as seizing the opportunity to demonstrate trust and build relationships both in face‐to‐face encounters and over the phone. Adopting a holistic approach and being accessible were also viewed as integral aspects of rapport building.

It's about trust; I think that's probably number one. So they feel they can come and ask questions and talk, and then it's really good if you can have such an open atmosphere so you can sort of air things out when something comes up… (Nurse 43)

Families of patients with substantial care needs or those visiting regularly were experienced as being easier to communicate with and build relationships with. Furthermore, the nurses found it simpler to engage with families who expressed concerns for their family members in need of HHC. Their perception was that engaging with families helped to increase the involvement of patients and their families. The nurses observed that families were often flexible and willing to actively participate in the care process.

#### 5.1.2 Exploring the Family's Communication Needs

According to the nurses' experiences, certain families exhibited a strong inclination towards communication, expressing a desire to engage in extensive conversations covering a wide range of topics to convey their emotions. In contrast, other families or individual family members could be less open to communicating, including being hesitant to ask questions, and could be reserved in expressing their feelings. Exploring the family's communication needs involved assessing and gaining an understanding of the family's information preferences. This included trying to understand or discover what lay beneath the surface, beyond what was apparent or instantly visible. It involved getting closer and gaining a more profound understanding of the family's needs.

I enjoy talking and involving everyone, trying to look beyond … how should I put it, behind the facade, so to speak…. (Nurse 38)

The nurses observed that family members sometimes found it more challenging to be open and honest in the presence of the family member who was receiving HHC, choosing to conceal sensitive matters in order to safeguard the sick family member. Additionally, communication with families could occasionally be challenging for the nurses when confidentiality prevented free discussion of the patient's overall situation.

It's like I say, it's very different. Some want to know more, and some want to know less. And there are things they shouldn't even know; it's about personal privacy too. So, you have to draw the line. somewhere (Nurse 35)

### 5.2 Struggling to Reach the Family

This category summarises the nurses' beliefs about potential obstacles to communication with families, including lack of understanding and challenges in reaching out to all family members.

#### 5.2.1 Lack of Understanding Hinders Effective Communication

The nurses believed that some families' lack of comprehension of the circumstances could make interactions with them challenging. Some families might not be aware of the patient's demands, or might not be aware of their own lack of understanding. According to the nurses, this lack of comprehension could be caused by certain families or family members occasionally thinking they knew more than the nurse did, or by certain families having strong beliefs and perceptions that were not always in the patient's best interests. The nurses believed that some families' lack of understanding stemmed from differences in opinion and perspectives regarding what would be effective and ineffective in the patient's care. This divergence was perceived to make communication and reaching a shared understanding more challenging.

When the daughters aren't in agreement, it's difficult, as they're the ones who organize things when both dad and mum have dementia… then it's crucial that the relatives are in agreement… otherwise, it's very challenging, and they do it all with good intentions and everything, but the understanding isn't there for… what might happen…. (Nurse 35)

It could be challenging for the nurses to comprehend and perceive the family's emotions, experiences and situation. Emphasis was placed on the importance of recognising the validity of all types of emotions and thoughts that the family could experience, including disappointment, fear and guilt. However, the nurses could not always detect these aspects from the families' mannerisms alone. To avoid misinterpretations and unclear understanding between the nurse and family members, it was considered crucial to uphold transparency and truthfulness in communication. Furthermore, they deemed it necessary to refrain from employing complicated medical terms, and instead to use language that was comprehensible to the patient.

Be open and honest and tell them that this is how it is and this is how it looks… not taking shortcuts just to soften the message, you don't need to… throw things in people's faces in a blunt way, but explain that this is how it is and… I think that's important… (Nurse 46)

#### 5.2.2 Challenges in Engaging the Whole Family

The nurses believed that some families divided the caregiving responsibilities among themselves. For example, one family member might oversee the patient's medical care while another managed the finances. This division of responsibilities could lead to challenges in identifying specific roles and whom to reach out to for practical matters. When the nurses, for instance, spoke to one or more family members and gave information that needed to be relayed to all members of the family, it could be challenging for the nurses to determine how and whether this information reached all the family members.

A family is dispersed in all directions, and when I call, it's obviously one person I talk to, but how he or she passes it on to the other siblings, I can't answer that. (Nurse 13)

The nurses experienced that it might be difficult to reach entire families, which could influence both the relationships between family members and those between nurses and families. The fact that family members occasionally did not speak to one another or held divergent perspectives could further contribute to the challenges.

One person thinks one way, and another thinks differently, and you don't quite get through, but then you just have to keep going and maintain that communication. (Nurse 35)

Given that communication and information between nurses and families were typically shared with the family member who lived closest, the nurses believed that distant family members were more frequently dissatisfied with the care because they had less opportunity to be physically present with the patient.

### 5.3 Empowering the Family to Empower Themselves

This category summarises the nurses' views on supporting self‐improvement by recognising the inherent value within families and mediating support.

#### 5.3.1 Acknowledging the Value of the Family

In the realm of nursing care, an understanding of the intrinsic value embedded within families formed a pivotal aspect in the context of caring for the patient. The nurses recognised the diverse roles assumed by family members, and acknowledged the varying degrees of engagement exhibited, ranging from more active participation to more passive involvement. Central to this was the acknowledgment that families played multifaceted roles with each member contributing uniquely to the familial dynamic.

I understand that within families, things look very different; they have different roles …. (Nurse 14)

An underlying tenet in this approach was the imperative to ‘think family’. By encouraging an acceptance of diverse emotions, the nurses conveyed the message that it was permissible for family members to experience a spectrum of feelings. Furthermore, the nurses recognised the needs of the relatives and provided a platform for their expression, underscoring the importance of attending not only to the patient but also to the broader familial context. In navigating the often patient‐centric focus of health care discussions, the nurses conscientiously redirected attention to the invaluable role played by family members. By acknowledging the broader impact of illness and health on the family, the nurses validated the experiences of relatives and underscored their integral part in the caregiving narrative.

The core [the family] around the patient, so it… it's very important… to achieve good nursing and care (Nurse 26).

#### 5.3.2 Conciliating Strengths

The nurses believed that their integral responsibilities included talking to the families when they were struggling, assisting them in overcoming challenges, and taking the time to listen when the families felt helpless. They also believed that various factors could influence families' perceptions of their environment and the patients, and that engaging in conversations with a nurse could enhance families' well‐being and empower them to provide mutual support. Nurses communicated the significance of fostering an atmosphere where familial bonds are strengthened through open dialogue and shared understanding. In this holistic approach, the nurse emerged as a facilitator of interconnectedness, striving to create an environment where families felt supported, understood and empowered in navigating the complexities of health care and well‐being.

Understanding each other, it's not about me but that they will get better communication with each other –patients, relatives, and families—overall, we can support that. (Nurse 22)

In their efforts to facilitate family support, the nurses strived to cultivate a climate that fostered comprehension within the family. Cognizant of these intricacies, they endeavoured to establish an atmosphere that promoted communication within the family constellation. This extended beyond the conventional nurse–patient relationship, emphasising the importance of facilitating discourse between family members themselves. The nurses demonstrated a keen interest in understanding the family members in order to identify the most effective ways to facilitate and assist them. Patients were perceived to have support within the health care organisation, although family members were occasionally encountered in states of confusion and invisibility. The nurses aspired to be a source from which family members could draw strength, support and guidance.

You make the family feel that they can handle a lot on their own, as long as they know where to turn or how to do things; you provide them with tools. (Nurse 23)

## 6 Discussion

The main findings of this study show the importance of building relationships through communication. Relationships were built through reciprocal communication between nurses and families, fostering a sense of familiarity and enhanced understanding between family and nurse as well as within families. This two‐way interaction led nurses to perceive that they were gaining profound insights into the family dynamics. The implications of these findings underscore the imperative for nurses to possess a comprehensive understanding of the supportive requisites of families, facilitated through well‐functioning communication. Consequently, the identification of families' discourse needs emerged as a crucial aspect of nursing practice. Moreover, the findings accentuate the inherent challenges that nurses may encounter in their endeavours to establish meaningful connections with families. These challenges underscore the nuanced nature of the nurse‐family dynamic, underlining the intricacies involved in reaching an optimal level of understanding and collaboration. Importantly, the essence of trust surfaced as a pivotal component within this discourse, emphasising the significance of cultivating a sense of trustworthiness and reliability in the nurse‐family relationship. Taken together, the findings indicate that healthy relationships between nurses and family members may be established through open conversation and by demonstrating respect and mutual trust.

The results indicate that nurses can enhance their understanding of families' unique needs by adopting a compassionate approach. This approach not only improves insight into family dynamics but also fosters a more comprehensive awareness and understanding of their situation. By fostering interactive relationships, conditions are created for families to develop trust in the nurse. This can be understood through Russell et al.'s (2021) description of the concept of trust within HHC. From the perspective of family members, the interpersonal aspects of their interactions are highlighted. Family members' trust in HHC providers involves the providers' technical and interpersonal skills, the promotion of regular and transparent communication, confidence in the providers' reliability for patient visits and ensuring adherence to the tasks outlined in the patient's care plan while addressing their needs (Russell et al. 2021). Higher levels of trust are associated with positive care outcomes, including increased satisfaction among families with the care provided (Boogaard et al. 2017). The current findings, along with existing literature, collectively underscore the central role of trust as a fundamental element. It is believed that such facilitation promotes increased understanding among participants. Additionally, family members in HHC may have strong desires for respect, recognition and understanding in their partnership with caregivers, emphasising the importance of feeling acknowledged and supported, and noting the impact of competent nurses in fostering a sense of safety (Søvde et al. 2019).

The nurses in the present study believed that patients' families had a considerable need for support in the form of dialogue with the nurses. However, they also described difficulties regarding communicating with families; these obstacles included the family's demand for privacy, passive involvement and struggles connecting with distant relatives. The nurses felt that some families' lack of understanding could make interactions challenging, as could families' lack of situational awareness and self‐awareness. Their strategies to mitigate these communication challenges included maintaining transparency and truthfulness to prevent misinterpretations and unclear understanding between nurses and family members. Furthermore, by fostering an acceptance of diverse emotions, the nurses communicated that it was acceptable for family members to undergo a range of feelings. The assertion that the life situation of family members is intricate and encompasses emotions that can be challenging to navigate, including contradictory feelings, is reinforced by Jarling et al. (2020). Through the adoption of shared responsibility, families' life situations can become more manageable and alleviate feelings of loneliness. This collaborative approach also offers a chance for a brief respite and a sense of freedom, whether through coordination with health care providers or through collaborating with other family members (Jarling et al. 2020).

According to our findings, the nurses felt that it was essential for them to be aware of and alert to the family's specific need for support through communication in their roles as professionals. Several of the key aspects described in the present study align with the description of a helping relationship given by Allande‐Cussó, Fernández‐García, and Porcel‐Gálvez (2022). The goal is to establish a relationship that constitutes a supportive connection with the patient and/or their family, grounded in interaction, communication, adherence to ethical values, acceptance and empathy. This relationship aims to foster introspection and behavioural change. Essential elements include functioning communication, active listening and the practice of respect (Allande‐Cussó, Fernández‐García, and Porcel‐Gálvez 2022). The findings in our study also highlight the importance of families for nursing, and indicate that a family systems nursing perspective is beneficial for all parties. This is confirmed by Bell (2021) and Shajani and Snell (2022), who describe family systems nursing as a holistic view of the family that promotes the nursing of the patient, as the family can be seen as a unit where each individual in the family affects each other as well as the patient. It is inevitable that nurses and families influence each other in the conversations and the relationship, and so nurses can use communication to alleviate the families' suffering (Shajani and Snell 2022).

### 6.1 Strengths and Limitations of the Work

One limitation of this study is the unequal gender distribution, as only one of our participants identified as male. The inclusion of additional male nurses might have contributed to a more comprehensive understanding of nurses' experiences in communication with families during HHC (cf. Graneheim and Lundman 2004); however, no additional male participants were available. Another potential limitation is that during the study period, a family systems nursing education intervention comprising health‐promoting family conversations was implemented concurrently within the municipal HHC. This might have influenced the high rate of participant engagement, as nearly all participants consented to be interviewed. The nurses' perceptions and experiences may have been partially informed by their participation in the intervention, which may have contributed to their deeper understanding of the significance of families in the caregiving process. However, it is essential to note that different continuous education initiatives are common in this setting, meaning that the situation during the study was not an unusual one. Furthermore, the questions posed during the interviews were broad, seeking insights into the nurses' overall experiences of communicating with families. The results of the analysis indicate that the participants' experiences were rich and wide ranging (cf. Polit and Beck 2022).

### 6.2 Recommendations for Further Research

Further research is necessary to get a better understanding of the mutual communication demonstrated in this study based on nurses' experiences. It would be interesting to study how nurse–family communication evolves longitudinally in home health care settings. Studying family members' experiences of communication with nurses in relation to having a close relative receiving HHC would add an important perspective. Similarly, the viewpoints of other health care personnel, such as home help providers who work closely with patients and their families, should also be explored in future research. By exploring the nuanced dynamics of nurses' communication with families in HHC, future research could delve into the impact of empathy and relationship‐building skills on family experiences. Studying effective strategies for trust‐building within the nurse–family dynamic would also contribute valuable insights to enhance communication and collaboration in this context. In addition, delving into the challenges and constraining beliefs that hinder nurses' engagement with families would be an intriguing avenue for further exploration. Understanding the barriers that nurses may face in establishing effective communication with families could offer valuable insights for developing strategies to overcome these challenges and foster more collaborative health care interactions.

### 6.3 Implications for Policy and Practice

This study has illuminated nurses' experiences of communicating with families in HHC. The results show the nurses' efforts to communicate with the families, and the contribution that this made to the nurses becoming familiar with the families and building a mutual relationship that invited participation and understanding. Collectively, the findings carry implications for nursing practice, underscoring the paramount importance of nurses' empathy, adept relationship‐building skills, communication, acknowledgement, appreciation of family members, the establishment of trust and providing crucial support. These findings highlight the foundational role nurses play in shaping positive interactions and outcomes within the context of family systems nursing in HHC settings. A learning intervention covering family systems nursing could be an appropriate measure to improve the requested approaches (cf. Bell 2021; Pusa, Isaksson, and Sundin 2021).

## 7 Conclusion

This research emphasises the critical importance of communication within HHC in the realm of nursing practice. It suggests that efficacious communication can cultivate a mutual relationship, thereby fostering a sense of trust. This gives the nurse the ability to ascertain the needs of the family and to extend the necessary support in an effective manner. To promote a family‐centred approach, it is suggested that nurses anchor their caregiving on the principles of family systems nursing theory. This approach may lead to intentional nursing interventions in communication that proactively involve the family in a reciprocal relationship. Consequently, specific communication training for nurses in home health care should be offered. In summary, the findings of this study provide valuable insights that can inform the development of future guidelines for HHC nursing.

## Author Contributions

All authors of this paper meet the authorship criteria according to the International Committee of Medical Journal Editors guidelines, and all authors have approved the final version of the manuscript.

## Ethics Statement

This study was approved by the Ethical Review Board in Sweden (2014‐235‐31Ö) and conducted in accordance with the Helsinki Declaration for Human Research (World Medical Association 2023).

## Conflicts of Interest

The authors declare no conflicts of interest.

## Data Availability

The authors have nothing to report.

## Appendix 1

**Table:**

Standards for reporting qualitative research (SRQR)^a^	Page/line no(s).
http://www.equator‐network.org/reporting‐guidelines/srqr/	
**Title and abstract**	
*Title*: Concise description of the nature and topic of the study Identifying the study as qualitative or indicating the approach (e.g., ethnography, grounded theory) or data collection methods (e.g., interview, focus group) is recommended	1/3
*Abstract*: Summary of key elements of the study using the abstract format of the intended publication; typically includes background, purpose, methods, results, and conclusions	1/5–25
**Introduction**	
*Problem formulation*: Description and significance of the problem/phenomenon studied; review of relevant theory and empirical work; problem statement	Background 3/74–81
*Purpose or research question*: Purpose of the study and specific objectives or questions	Background 3/83–84
**Methods**	
*Qualitative approach and research paradigm*: Qualitative approach (e.g., ethnography, grounded theory, case study, phenomenology, narrative research) and guiding theory if appropriate; identifying the research paradigm (e.g., postpositivist, constructivist/ interpretivist) is also recommended; rationale^b^	Design 3/87–90
*Researcher characteristics and reflexivity*: Researchers’ characteristics that may influence the research, including personal attributes, qualifications/experience, relationship with participants, assumptions, and/or presuppositions; potential or actual interaction between researchers’ characteristics and the research questions, approach, methods, results, and/or transferability	Rigor and reflexivity 5/140–147
*Context*: Setting/site and salient contextual factors; rationale^b^	Study Setting and Recruitment 3/64
*Sampling strategy*: How and why research participants, documents, or events were selected; criteria for deciding when no further sampling was necessary (e.g., sampling saturation); rationale^b^	Study Setting and Sampling 3/92–98 Inclusion Criteria 4/99–101
*Ethical issues pertaining to human subjects*: Documentation of approval by an appropriate ethics review board and participant consent, or explanation for lack thereof; other confidentiality and data security issues	Ethical consideration 5/133–139
*Data collection methods*: Types of data collected; details of data collection procedures including (as appropriate) start and stop dates of data collection and analysis, iterative process, triangulation of sources/methods, and modification of procedures in response to evolving study findings; rationale^b^	Data collection 4/102–111
*Data collection instruments and technologies*: Description of instruments (e.g., interview guides, questionnaires) and devices (e.g., audio recorders) used for data collection; if/how the instrument(s) changed over the course of the study	Data collection 4/102–111
*Units of study*: Number and relevant characteristics of participants, documents, or events included in the study; level of participation (could be reported in results)	Characteristics of participants 5–6/161–168
*Data processing*: Methods for processing data prior to and during analysis, including transcription, data entry, data management and security, verification of data integrity, data coding, and anonymization/de‐identification of excerpts	Data collection 3/102–111
*Data analysis*: Process by which inferences, themes, etc., were identified and developed, including the researchers involved in data analysis; usually references a specific paradigm or approach; rationale^b^	Data analysis 4/112–121
*Techniques to enhance trustworthiness*: Techniques to enhance trustworthiness and credibility of data analysis (e.g., member checking, audit trail, triangulation); rationale^b^	Rigor and reflexicity 5/140–147 Strengths and Limitations of the Work 12/374–386
**Results/findings**	
*Synthesis and interpretation*: Main findings (e.g., interpretations, inferences, and themes); might include development of a theory or model, or integration with prior research or theory	Findings 5–9/160–303
*Links to empirical data*: Evidence (e.g., quotes, field notes, text excerpts, photographs) to substantiate analytic findings	Findings 5–9/160–303
**Discussion**	
*Integration with prior work, implications, transferability, and contribution(s) to the field*: Short summary of main findings; explanation of how findings and conclusions connect to, support, elaborate on, or challenge conclusions of earlier scholarship; discussion of scope of application/generalizability; identification of unique contribution(s) to scholarship in a discipline or field	Discussion 10–13/305–419
*Limitations*: Trustworthiness and limitations of findings	Strengths and Limitations of the Work 12/374–386
**Other**	
*Conflicts of interest*: Potential sources of influence or perceived influence on study conduct and conclusions; how these were managed	N/A
*Funding*: Sources of funding and other support; role of funders in data collection, interpretation, and reporting	N/A

^a^ The authors created the SRQR by searching the literature to identify guidelines, reporting standards, and critical appraisal criteria for qualitative research; reviewing the reference lists of retrieved sources; and contacting experts to gain feedback. The SRQR aims to improve the transparency of all aspects of qualitative research by providing clear standards for reporting qualitative research.

^b^ The rationale should briefly discuss the justification for choosing that theory, approach, method, or technique rather than other options available, the assumptions and limitations implicit in those choices, and how those choices influence study conclusions and transferability. As appropriate, the rationale for several items might be discussed together.
